# Evaluation of the risk of cervical cancer in patients with Multiple Sclerosis treated with cytotoxic agents: A cohort study

**Published:** 2018-04-04

**Authors:** Rozita Doosti, Mansoureh Togha, Abdorreza Naser Moghadasi, Aida Aghsaie, Amir Reza Azimi, Saeideh Khorramnia, Zahra Moinfar, Fereshteh Ensani, Mohammad Hossein Harirchian, Alireza Minagar, Mohammad Ali Sahraian

**Affiliations:** 1 Multiple Sclerosis Research Center, Neuroscience Institute, Tehran University of Medical Sciences, Tehran, Iran; 2 Department of Neurology, Sina Hospital, Tehran University of Medical Sciences, Tehran, Iran; 3 Department of Pathology, Imam Khomeini Hospital, Tehran University of Medical Sciences, Tehran, Iran; 4 Iranian Center for Neurological Research, Imam Khomeini Hospital, Tehran University of Medical Sciences, Tehran, Iran; 5 Department of Neurology, Louisiana State University Health Sciences Center, Shreveport, Louisiana, USA

**Keywords:** Pap Smear, Multiple Sclerosis, Cytotoxic Agents

## Abstract

**Background:** Since most patients with relapsing-remitting multiple sclerosis (RRMS) are women, the present study aimed to determine whether treatment of patients with MS by cytotoxic agents is associated with an increased risk of cervical dysplasia. Cancer screening is often neglected in the chronic diseases such as MS, so more attention in this field was needed. Decreasing morbidity and mortality due to cervical cancer is the most important goal of screening in female MS patients especially in child bearing age. Thus, it can be said that this is the first study which investigated this important issue.

**Methods:** A total of 129 individuals participated in this cohort study. They were assigned into 3 groups including 43 patients with MS who were treated with cytotoxic drugs, 43 patients with MS on immunomodulators, and 43 normal healthy controls. Pap smears were performed following standard methods and the results obtained from the three groups were compared by statistical analysis. Demographic data, Expanded Disability Status Scale (EDSS), and Pap smear changes were analyzed by SPSS software.

**Results:** The most commonly detected abnormality in all examined patients and healthy controls was inflammation. Five patients with MS who were treated with cytotoxic agents revealed benign cellular changes (BCC) in their Pap smear that were statistically significant in comparison with other groups (P = 0.03). Patients who took Mitoxantrone presented BCC more than other groups [Odds ratio (OR) = 9.44, 95% confidence interval (CI): 1.46-60.70]. There was no significant difference between mean duration of MS diagnosis (P = 0.12), mean duration of previous MS treatments (P = 0.25), and mean duration of current MS treatments (P = 0.21) in patients with BCC compared to normal healthy controls or inflammatory change.

**Conclusion:** According to the results of present study, BCC is more frequently observed in patients with MS who were treated with cytotoxic agents with immunosuppressive effect. Since BCC is a ‘premalignant condition’, the authors suggest that mandatory annual Pap smear should be performed for patients with MS who are treated with cytotoxic agents irrespective of their age in order to detect early signs of malignancy.

## Introduction

Multiple sclerosis (MS) is a chronic inflammatory demyelinating and neurodegenerative disease of human central nervous system (CNS), which often results in the development of various neurological manifestations. Cytotoxic agents such as Mitoxantrone, Mycophenolate mofetil, Cyclophosphamide, and Azathioprine are prescribed to prevent severe relapses by inducing immunosuppression in certain patients with secondary progressive MS (SPMS) or progressive relapsing MS (PRMS).^1^^-^^3^ Azathioprine is occasionally prescribed as an add-on therapy to decrease the number of clinical relapses.^4^ Inhibition of tumor necrosis factor-α (TNF-α), interlukin-1β (IL-1β), and monocyte chemotactic protein-1 (MCP-1) production may be their mechanism of action.^5^ Cytotoxic agents which cause profound immunosuppression in these patients possess serious adverse effects which include secondary malignancy and premalignant conditions involving various organs. Acute myeloid leukemia,^6^ bladder cancer,^7^ and solid tumors^8^ are life-threatening malignancies which have been reported to be the result of prolonged utilization of these cytotoxic agents. However, as far as the authors of the present study are concerned, the risk of other types of malignancies or pre-cancerous conditions in this group of patients has not been fully assessed and determined.

Most patients with relapsing forms of MS are women of child bearing age, and the incidence of certain premalignant conditions such as cervical dysplasia may be higher in these patients and easily ignored by the clinicians. Cancer screening is often neglected in the chronic diseases such as MS, so more attention in this field was needed. Decreasing morbidity and mortality due to cervical cancer is the most important goal of screening in female MS patients especially in child bearing age. Thus, it can be said that this is the first study which investigated this important issue.

In this regard, this study aimed to explain cervical dysplasia or other Pap smear (Pap) abnormalities in female MS patients receiving immunomodulators (Interferon-β 1a and b) or immunosuppressive medications.

## Materials and Methods

This cohort study was approved by Ethics Committee of Tehran University of Medical Sciences (approval number IR.TUMS.REC.1395.2797), and was performed in Sina hospital, Multiple sclerosis Research Center, Neuroscience Institute, Tehran University of Medical Sciences, Tehran, Iran.

During three years, since Jan 2011 to Jan 2013, 129 individuals including three groups, 43 patients with relapsing-remitting (RRMS), 43 patients with SPMS, and 43 healthy individuals, were recruited in this study. All patients received their treatments without any intervention during this time.

Multiparty women aged between 18 to 70 years old, diagnosed with RRMS or SPMS based on McDonald’s criteria (2010), within expanded disability status scale (EDSS) between 0-5, and having only one sex partner participated in this study.

Patients who were identified as current smokers, had history of using oral contraceptive pills (OCPs) or intrauterine device, diagnosed with active infection of sexually transmitted diseases (STDs) including human papillomavirus (HPV), herpes simplex virus (HSV), chlamydia trachomatis, trichomonas vaginalis, etc., were pregnant, and had history of exposure to diethylstilbestrol were excluded from the study.

All eligible patients had to sign informed consent for enrollment. In this study, a neurologist performed neurologic exams including EDSS evaluation. Three general practitioners (GPs) evaluated patients’ baseline characteristic information (such as age, marital status, parity, number of sex partners, smoking status, using OCP, STDs, pregnancy, past medical history, and previous MS medications) and performed Pap smear on patients. Simple Pap smear examinations were performed under standard conditions.

Three groups were enrolled in this study: the first group consisted of 43 patients with RRMS who were under treatment by immunomodulators (Interferon-β 1a and b) within 3 years before conducting this study. 

**Table 1:** Baseline characteristic information of participants

**Variable**	**Immunomodulator ** **group**	**Immunosupp** **ressive group**	**Healthy people ** **(control group)**	**P**	**Statistical ** **test**
Age (year) (mean ± SD)	38.84 ± 8.29	39.21 ± 8.55	40.23 ± 9.03	0.740	ANOVA
Duration of MS diagnosis (months) (mean ± SD)	35.28 ± 22.73	48.81 ± 22.17	-	0.006	T
Duration of previous MS treatments (months) (mean ± SD)	14.82 ± 5.65	16.86 ± 9.95	-	0.001	T
Duration of current MS Treatments (months) (mean ± SD)	26.35 ± 17.24	18.91 ± 15.41	-	0.037	T
EDSS	1.13 ± 0.90	3.89 ± 0.44	-	0.001	T

MS: Multiple Sclerosis; EDSS: Expanded Disability Status Scale

The second group consisted of 43 patients with SPMS who were under treatment by immunosuppressive medications (including Mitoxantrone, Methotrexate, Azathioprine, cyclophosphamide, and Cellcept) within 3 years before conducting this study. The first and the second groups were examined by three GPs for performing Pap smear. 

The third group consisted of 43 individuals (the control group included healthy individuals) whose Pap smears results were sent to the same laboratory during these three years. The results obtained from this group were evaluated for determining Pap smears differences between normal population and the two MS patients group.

To avoid any bias, the researchers labeled all samples with participants' number, initials, and age. All samples were reviewed by a professional pathologist in Danesh laboratory who gave her opinion about the grade of Pap smears according to the revised Bethesda system.^9^ Statistical analysis was performed using SPSS software (version 18, SPSS Inc., Chicago, IL, USA). Demographic data, EDSS, and Pap smear changes were analyzed. The study reported mean values of baseline characteristic information to describe numeric variables.

## Results

A total of 129 individuals participated in this study. The baseline characteristic information of participants is demonstrated in table 1.

The mean ± standard deviation (SD) age of participants in this study in the immunomodulator group, the immunosuppressive group, and the control group were 38.84 ± 8.29, 39.21 ± 8.55, and 40.23 ± 9.03 years, respectively. There was no statistically significant difference between the mean ± SD age of participants in these three groups according to one-way ANOVA (P = 0.74), as shown in table 1.

The mean ± SD age of all participants was 39.43 ± 8.58 and the minimum and the maximum ages were 24 and 66 years, respectively.

The main current MS treatments received by the patients before enrollment in the study included Avonex (n = 23, 53.5%) in the first group, and Mitoxantrone (n = 20, 46.5%) in the second group. Moreover, the main previous MS treatments received by the patients before enrollment included nothing (n = 32, 74.4%) in the first group and Betaferon (n = 17, 39.5%) in the second group. The detailed information of each group is presented in table 2.

**Table 2:** Current and previous multiple sclerosis (MS) treatments of patients with MS in this study

**Patients with MS**	**Drugs name**	**Current MS treatments [n (%)]**	**Previous MS treatments [n (%)]**
Immunomodulator group	None	0 (0)	32 (74.4)
	Avonex	23 (53.5)	11 (25.6)
	Betaferon	8 (18.6)	0 (0)
	Rebif	12 (27.9)	0 (0)
Immunosuppressive group	None	0 (0)	0 (0)
	Avonex	0 (0)	4 (9.4)
	Betaferon	0 (0)	17 (39.5)
	Rebif	0 (0)	11 (25.6)
	Azathioprine	3 (7.0)	5 (11.6)
	Methotrexate	1 (2.3)	1 (2.3)
	Cellcept	19 (44.2)	0 (0)
	Mitoxantrone	20 (46.5)	5 (11.6)

MS: Multiple Sclerosis

**Table 3:** Pap smear results and age distribution in three groups of study

**Groups**	**Pap smear results**		
	**Normal [n (%)]**	**Inflammatory changes [n (%)]**	**BCC [n (%)]**
Immunosuppressive group	18 (41.9)	20 (46.5)	5 (11.6)
Immunomodulator group	22 (51.2)	21 (48.8)	0 (0)
Healthy people (control group)	23 (53.5)	20 (46.5)	0 (0)

BCC: Benign Cellular Changes

The mean ± SD time of MS diagnosis were 35.28 ± 22.73 and 48.81 ± 22.17 months in the first and the second groups, respectively. The mean ± SD EDSS at time of performing samples were 1.13 ± 0.90 and 3.89 ± 0.44 in the first and the second groups, respectively (Table 1). The maximum time of exposure to the current MS treatments in the first group was 60 months compared with 84 months in the second group. The minimum time of exposure to the current MS treatments in the first group was 4 months and in the second group was 1 months.

Mean ± SD duration of current MS treatment in the immunomodulator group was 23.26 ± 19.72 months for Avonex, 33.50 ± 16.72 months for Betaferon, and 27.50 ± 10.99 months for Rebif. Mean ± SD duration of current MS treatment in the immunosuppressive group was 24.67 ± 11.01 months for Azathioprine, 36.00 ± 0 months for Methotrexate, 22.89 ± 20.18 months for Cellcept, and 13.40 ± 7.59 months for Mitoxantrone.

As showed in table 3, on Pap examination, inflammation was the most commonly observed abnormality detected in almost half of the cases in each group (n = 63, 48.8%). That is to say, 46.5% (20 cases) of patients with MS who were treated with immunomodulatory drugs, 48.8% (21 cases) of patients with MS who were treated with immunosuppressive drugs, and 46.5% (20 cases) of the healthy controls had inflammation on their Pap examinations. Chi-square test showed a statistically significant difference in this regard between these three groups (P = 0.03). Among patients who were treated with immunosuppressive agents, five patients (11.6%) were reported to have benign cellular changes (BCC) in their Pap smears which suggested a statistically significant difference according to Fisher's exact test (P = 0.03). Characteristic information of the patients with BCC is revealed in table 4.

After categorizing the Pap smear results into two groups (as BCC and inflammatory or normal), in binary logistic regression, the relationship between using immunosuppressive drugs (immunomodulatory and immunosuppressive) and Pap smear results [odds ratio (OR): 2.41, 95% confidence interval (CI):-0.24-5.06, P = 0.07] tended to be significant. Moreover, there was a significant relationship between immunosuppressive drugs and Pap smear results (P = 0.01) after adjusting for potential confounders including age and EDSS. Patients who took Mitoxantrone presented BCC more than other groups (OR = 9.44, 95% CI: 1.46-60.70) (Table 4).

**Table 4:** Characteristic information of patients with benign cellular changes (BCC)

**Variable**	**Value**	**OR (95% CI)**
Age (year) (mean ± SD)	36.20 ± 6.06	-
Duration of MS diagnosis (months) (mean ± SD)	57.60 ± 14.15	-
Duration of previous MS treatments (months) (mean ± SD)	18.00 ± 10.39	-
Previous MS treatments [n (%)]		-
Azathioprine	3 (60)	
Mitoxantrone	2 (40)	
Duration of current MS treatments (months) (mean ± SD)	13.60 ± 5.86	-
Current MS treatments [n (%)]		9.441 (1.468-60.704)
Mitoxantrone	3 (60)	
Azathioprine	1 (20)	
Cellcept	1 (20)	
EDSS (mean ± SD)	4.50 ± 0.00	-

SD: Standard deviation; OR: Odds ratio; CI: Confidence interval; MS: Multiple Sclerosis; EDSS: Expanded Disability Status Scale

**Table 5:** Mean comparison of some predictors based on BCC status

**Variable**	**BCC**	**Normal or ** **Inflammatory change**	**P (T test)**
Age (year) (mean ± SD)	36.20 ± 6.05	39.55 ± 8.66	0.393
Duration of MS diagnosis (months) (mean ± SD)	57.60 ± 14.15	41.08 ± 23.50	0.125
Duration of previous MS treatments (months) (mean ± SD)	18.00 ± 10.39	10.83 ± 13.87	0.261
Duration of current MS treatments (months) (mean ± SD)	13.60 ± 5.85	23.18 ± 16.98	0.214
EDSS (mean ± SD)	4.50 ± 0	2.38 ± 1.52	0.002

BCC: Benign cellular changes; MS: Multiple Sclerosis; EDSS: Expanded Disability Status Scale; SD: Standard deviation

The mean ± SD of EDSS in patients with and without BCC were 4.50 ± 0.00 and 2.38 ± 1.52, respectively; the results of t-test showed that the difference was statistically significant (P < 0.01). In addition, the mean ± SD of age in patients with and without BCC were 36.20 ± 6.05 and 39.55 ± 8.66 years, respectively; the difference was not statistically significant (P = 0.39). Furthermore, there was no significant difference between mean of duration of MS diagnosis (P = 0.12), duration of previous MS treatments (P = 0.26) and duration of current MS treatments (P = 0.21) in patients with BCC compared to normal healthy controls or inflammatory change (Table 5).

## Discussion

In this study, it was shown that patients with MS who took immunosuppressive drugs as current treatment had more chance to present BCC in their Pap smear results. Mitoxantrone had more relation on presenting BCC among immunosuppressive drugs. Furthermore, previous treatments in these patients were immunosuppressive drugs as well. MS is a relatively common disease in young women, and the mean age and duration of MS diagnosis in this study revealed importance of Pap examinations in them. It should be noted that in the chronic diseases such as MS, other aspects of the patient’s life is under the influence of the main disease and cancer screening is neglected usually in them. 

The rate of cervical dysplasia or other Pap abnormalities has not been studied in patients with MS previously, as far as the authors of the present study are concerned. Thus, it can be said that this is the first study which investigated this important issue. The findings of the present study revealed an increase in BCC rate in patients with MS who were treated with immunosuppressive drugs. Although BCC was previously mentioned as a benign process caused by infection, inflammation, or reactive cellular changes; nowadays, there is an intention to separate BCC from those Pap changes recognized as “Within Normal Limits” (WNL).^9^ It has been shown that some patients, for whom BCC was reported in Pap examinations, had cervical intraepithelial neoplasia (CIN) in their biopsies.^10^^,^^11^ As BCC was detected only in patients who were immunocompromised due to cytotoxic medications, and not in the other two groups, this should be considered as a complication of cytotoxic agents. Higher rates of cervical dysplasia in patients with other diseases requiring immunosuppressive medications have been reported previously.^12^^-^^14^ Bernatsky, et al., for instance, studied 1015 patients with lupus and evaluated the incidence of abnormal Pap smears and some other risk factors such as smoking status, history of STDs, and usage of OCP.^13^ They found that 13.3% of their patients had abnormal Pap smears. Although several different factors could increase the risk of cervical dysplasia in these patients, the authors concluded that immunosuppressive agents might be a trigger for facilitating HPV to induce abnormal cell differentiation and predispose those patients to premalignant conditions.^13^

Although World Health Organization (WHO) suggested guideline for cervical cancer screening (especially in precancerous lesions), American Society for Colposcopy and Cervical Pathology (ASCCP) recommends Pap test every 3 years for initializing, and co-testing (including HPV testing and Pap test) every 5 years for women with negative results of both initial tests.^15^

In another study, Bernatsky, et al., reviewing the evidence obtained from systemic lupus erythematous (SLE) patients’ susceptibility to different malignancies, concluded that the impairment of HPV clearance and the effect of immunosuppressive medications may be two of the most crucial causes of cervical dysplasia.^12^

Dreyer, et al. revealed that HPV-dependent malignancies including cervical dysplasia, and anogenital and oropharyngeal cancers have higher rates among SLE patients.^14^ Furthermore, HPV may be a predisposing factor for non-melanoma skin cancer,^10^ the incidence of which was reported in previous studies to be higher among immunocompromised patients.^14^

Recent studies in immunocompromised women suffering from SLE revealed that these patients have increased chance of presenting more premalignant lesions (with or without HPV infection) than the general population.^16^^-^^20^ Although recent study revealed special guideline for screening and follow-up of cervical cancer in immunosuppressant women such as women with SLE, irritable bowel syndrome (IBS) and other immunocompromised women, perhaps MS may be different from other diseases.^21^

Despite the lack of co-testing for evaluation of our patients, our study provided special data on MS immunocompromised women which may have different condition for presenting abnormal cervical cytology. In the present study, patients with MS with different levels of immunosuppression revealed precancerous cervical lesions which may be different from findings of patients with SLE in other studies, so it would be of interest for a future study to include different type of diseases treated with immunosuppressive drugs for evaluating role of disease on presenting abnormal cervical cytology.

According to the mentioned evidences, HPV may play an important role in developing BCC and other probable cervical dysplasia in patients with MS who receive immunosuppressive medications; it should be noted that clearance defect of HPV is the most significant cause of cervical dysplasia and other Pap smear abnormalities in these patients. Cytotoxic agents change the nature of immunity and immunological defense against different infections; thus, HPV can induce abnormal cell differentiation and lead to premalignant and malignant conditions.

CIN is a significant predisposing factor for cervical cancer. There have been several factors which make women more likely to suffer from these threatening conditions including infections, smoking, status, and history of infertility.^22^ Moreover, similar to normal individuals, patients with MS can be affected by these predisposing factors. In addition, according to the results obtained from the present study, immunosuppressive agents should be considered as additional risk factors for cervical dysplasia in patients with MS. Thereafter, the authors highly recommend that annual Pap smear should be done for patients with MS receiving cytotoxic medications. This strategy may protect them against cervical malignancy through early diagnosis.

Moreover, duration of MS diagnosis and duration of (previous or current) treatments may have a role in BCC change in Pap examination, which necessitates having more narrow observation for performing annual Pap smears.

One of the limitations of this study is low use of Novantrone in the presence of other drugs which have a high potential and are increasingly used for the treatment of MS. Among these drugs are Fingolimod, Tysabri, Rituximab, Alemtuzumab, and Eculizumab. These drugs question the use of drugs that have various side effects such as Novantrone. Although this limitation is one of the drawbacks of this study, it should be noted that the potential side effects of the drugs such as Rituximab, alemtuzumab, or Eculizumab are still unknown. These drugs have significant effects on the patient's immune system which may cause side effects such as those mentioned in the present study. Moreover, patients with MS are mostly young individuals among them women have considerably the most ratio. This increases the risk of getting such diseases in young women during treatment that regarding their low age is long. Thereafter, in addition to emphasizing on paying attention to women’s disorders, the present study proposed a framework for further studies.

Moreover, Szarewski and Sasieni estimated that 92.5% of low-grade abnormalities regressed within 24 months and 6.5% progressed in young women.^23^ Ronco, et al. suggested that HPV screening leads to over-diagnosis of regressive low grade lesions in Pap tests of young women.^24^

Another aspect that must be taken into account is that the abnormal lesions detected in the most patients may regress in follow-up observations; so, HPV testing in combination with Pap test (co-testing) and having numerous follow-up samples of our patients is another limitation of our study.

The present study had some other limitations including small number of participants. Besides, the study could not detect which immunosuppressive drug is more associated with cervical dysplasia. Moreover, the study could not judge about the risk of cytotoxic monotherapy versus poly therapy, and find a cut-off cumulative dose of treatment for increased risk of cervical dysplasia. These issues should be addressed in other large multi-center cohort studies in future.

## Conclusion

According to the results of the present study, BCC is more frequently observed in patients with MS who were treated with cytotoxic agents with immunosuppressive effect. Since BCC is a ‘premalignant condition’, the authors suggest that mandatory annual Pap smear should be performed for patients with MS who are treated with cytotoxic agents irrespective of their age in order to detect early signs of malignancy.

## Notes:

**How to cite this article:** Doosti R, Togha M, Naser Moghadasi A, Aghsaie A, Azimi AR, Khorramnia S, et al. Evaluation of the risk of cervical cancer in patients with Multiple Sclerosis treated with cytotoxic agents: A cohort study. Iran J Neurol 2018; 17(2): 64-70
